# Effect of STAT5 silenced by siRNA on proliferation apoptosis and invasion of esophageal carcinoma cell line Eca-109

**DOI:** 10.1186/1746-1596-8-132

**Published:** 2013-08-05

**Authors:** Qian Yang, Min Li, Tao Wang, Hong Xu, Wenqiao Zang, Guoqiang Zhao

**Affiliations:** 1 Medical Examination Center, The First Affiliated Hospital of Henan University of TCM, Zhengzhou, People’s Republic of China; 2 College of Basic Medical Sciences, Zhengzhou University, Zhengzhou, People’s Republic of China; 3 Department of Hemato-tumor, The First Affiliated Hospital of Henan University of TCM, Zhengzhou, People’s Republic of China; 4 Henan Tumor Institute, Zhengzhou, People’s Republic of China

**Keywords:** STAT5, siRNA, Proliferation, Cell cycle, Apoptosis

## Abstract

**Background:**

STAT is the backward position of cytokine and growth factor receptors in the nucleus, STAT dimers could bind to DNA and induce transcription of specific target genes. Several lines of evidence support the important roles of STAT, especially STAT5, in carcinogenesis. The overexpression of STAT 5 is related to the differentiation and apoptosis of tumor cells. However, the role of STAT5 in esophageal squamous cell carcinoma remains unclear.

**Methods:**

The siRNA vectors aiming to STAT5 gene were constructed. STAT5 siRNA was transfected into Eca-109 cells by Lipofectamine™2000. Expression of STAT5、Bcl-2 and Cyclin D1 were analyzed by Western blot and RT-PCR. Eca-109 cells proliferation was determined by MTT. Eca-109 cell cycle and apoptosis were detected by the flow cytometry. Boyden chamber was used to evaluate the invasion and metastasis capabilities of Eca-109 cells.

**Results:**

The double strands oligonucleotide of siRNA aiming to STAT5 was successfully cloned into the pRNAT-U6.1 vector, and the target sequence coincided with the design. RT-PCR and Western blotting detection demonstrated that the expression levels of STAT5、Bcl-2 and Cyclin D1 gene were obviously decreased in Eca-109 cells transfected with STAT5 siRNA. STAT5 siRNA could suppress the proliferation of Eca-109 cells. The proportion of S and G2/M period frequency was significantly decreased (p < 0.05). The proportion of G0/G1 period frequency was significantly increased (p < 0.05). The average amount of cells penetrating Matrigel was significantly decreased (p < 0.05).

**Conclusions:**

STAT5 silenced by siRNA could induce the apoptosis and suppress the proliferation、invasion and metastasis of esophageal carcinoma cell line Eca-109, which indicated STAT5 might be a novel therapeutic strategy for the human ESCC.

**Virtual slides:**

The virtual slide(s) for this article can be found here: http://www.diagnosticpathology.diagnomx.eu/vs/1351913072103000

## Introduction

The STAT (signal transducer and activator of transcription) family of proteins includes 7 members (STAT −1、2、3、4、5a、5b and 6) encoded by distinct genes in mammalian cells. The STAT family members are latent cytoplasmic transcription factors that are activated in response to extracellular signaling proteins, including growth factors, cytokines, hormones, and peptides [1-5]. STAT is the backward position of cytokine and growth factor receptors in the nucleus, STAT dimers bind to DNA and induce transcription of specific target genes [6-9]. The overexpression of STAT 5 is related to the differentiation、apoptosis and new capillaries of tumor cells [10-14]. In this study, We silenced STAT5 by siRNA to explore the effect on proliferation 、apoptosis and invasion of esophageal carcinoma cell line Eca-109, which gave a certain target in gene therapy of Esophageal Carcinoma.

## Materials and methods

### Main materials

siRNA vectors pRNAT-U6.1/Neo (GeneScript Corp,China); BamHI 、HindIII and T4 DNA ligase(Promega Corp, USA);the first antibody of STAT5, Bcl-2, Cyclin D1,GAPDH(Santa Cruz Corp, USA); RPMI 1640; LipofectAmine™2000, G418(Invitrogen, USA); MTT、DMSO、Trypsin、PI、RNAase(Sigma Corp, USA); Esophageal carcinoma cell line Eca-109 were obtained from the basic medical college of Zhengzhou University.

### Design the STAT5 gene target oligonucleotide

Abide by the principle of design siRNA fragment and the target oligonucleotide was designed with the help of the GenScript siRNA Target Finder software of GenScript company, which the following web address provided: https://www.genscript.com/ssl-bin/app/rnai The target sequences located in 2851nt-2869nt (GGCAGTGAGTTTCGTGAAG).

### The construction of siRNA vectors

Two couples of hairpin sample DNA oligonycleotides (2851-1、2851-2 and Con-1、Con-2)were annealed to produce dsDNA(siSTAT5 and siCon). Then the dsDNA was inserted into the BglII and HindIII site of thepRNAT-U6.1/Neo vector.2 × reaction buffer 5 μL, sticking end linear pRNAT-U6.1 /Neo vectors 1 μL, T4 DNA ligase 1 μL, dsDNA(siSTAT5 and siCon)3 μL, All of them were for a whole night at 4°C. The recombinant vectors were transformed into Escherichia coli DH5α. The pRNAT-U6.1/Neo-siSTAT5 and pRNAT-U6.1/Neo-siCon vectors were constructed after the analyzsis of consequense.

### The transfection of Eca-109 cells with Lipofectamine™2000

When the density of plasmids was 2 μg/ml, the pRNAT-U6.1/Neo-siSTAT5 and pRNAT-U6.1/Neo-siCon were transfected into Eca-109 cells, as the experiment group and the siRNA control group. According to the manufacturer’s protocol of Lipofectamine™2000, and the cells were cultured after 6 h. The experiments were performed independently four times.

### RT-PCR

The GAPDH was used as the internal reference, then the primers and probes were designed according to the software of Primer Express 3.0(ABI Corp), which all are synthetized by Shanghai bioengineering company (Table 1). Total RNA was isolated from the cells using Trizol extraction kit according to the manufacturer’s protocol. RT-PCR was performed with the apparatus of ABI Step One Plus PCR. The ratio of the copies of the detected genes (STAT5、Bcl-2、Cyclin D1)to the copies of GAPDH was the relative expression quantity. The experiments were performed independently four times.

**Table 1:** RT-PCR probes

**Names**	**Probes**	**Sequence**
STAT5	Forward Primer	5’ GCTGGAAGCCTTGCTGAT 3’
	Reverse Primer	5’ TCCTCAAACGTCTGGTTGATC 3’
	Probe	5’ FAM-TGTCCCAGAAACACCTC–TAMRA 3’
Bcl2	Forward Primer	5’ CATGTGTGTGGAGAGCGTCAA 3’
	Reverse Primer	5’ GCCGGTTCAGGTACTCAGTCAT 3’
	Probe	5’ FAM-TGGACAACATCGCCCTGT–TAMRA 3’
Cyclin-D1	Forward Primer	5’ GTGGCCTCTAAGATGAAGGA 3’
	Reverse Primer	5’ GGTGTAGATGCACAGCTTCT 3’
	Probe	5’ FAM-ACCATCCCCCTGACGGC–TAMRA 3’
GAPDH	Forward Primer	5’ GGTGGTCTCCTCTGACTTCAACA 3’
	Reverse Primer	5’ CCAAATTCGTTGTCATACCAGGAAATG 3’
	Probe	5’FAM-CGACACCCACTCCTCCACCTTTGACGC–TAMRA 3’

The primers and probes were designed according to the software of Primer Express 3.0(ABI Corp), which all were synthetized by Shanghai bioengineering company, China.

### Western blot

Cells were lysed for total protein extraction. The protein concentration was determined by the BCA method (KeyGEN, China), and 30 μg of protein lysates were subjected to SDS-PAGE. The electrophoresed proteins were transferred to nitrocellulose membranes (Whatman, USA), which were blocked in 5% non-fat milk and incubated overnight at 4°C with diluted first antibodies. Membranes were then incubated with HRP-conjugated secondary antibody (1:2,500, Santa Cruz, USA). After washing with PBST buffer (PBS containing 0.05% Tween-20), membranes were probed using ultra-enhanced chemiluminescence western blotting detection reagents. GAPDH was used as the internal reference.

### MTT assay

The experimental groups of cells in the logarithmic phase of growth were seeded in 96-well plates at a cell density of 0.4 × 10^4^/well. For six consecutive days, 20 μl of MTT (5 mg/ml) was added to the corresponding well, cells were incubated at 37°C for an additional 4 h, and the reaction was stopped by lysing the cells with 200 μl of DMSO for 20 min. Optical density was measured at 590 nm The experiments were performed independently four times.

### The flow cytometry detects the cell cycle

For cell cycle analysis by flow cytometry (FCM), cells in the logarithmic phase of growth were harvested by trypsinization, washed with PBS, fixed with 75% ethanol overnight at 4°C and incubated with RNase at 37°C for 30 min. Nuclei were stained with propidium iodide for 30 min. A total of 10^4^ nuclei were examined in a FACSCalibur Flow Cytometer (Becton Dickinson, Franklin Lakes, NJ, USA). The experiments were performed independently four times.

### Cell apoptosis assay

Every group cells were harvested and diluted with PBS twice. Then 5 μL of FITC-labeled enhanced-annexinV and 5 μl 20 μg/ml of propidium iodide were added to 100 μl cell . Upon incubation in the dark for 15 min at room temperature, samples were diluted with 400 μl PBS. Flow cytometry was carried out on a FACS can instrument. The result was analysed by random software. The experiments were performed independently four times.

### Cell invasion assay

The invasion ability of Eca-109 cells was assayed using Transwells (8-μm pore size, Corning Costar Corp). Transwells filters were coated with matrigel (3.9 μg/μl, 60-80 μl) on the upper surface of the polycarbonic membrane (6.5 mm in diameter, 8 μm pore size). Eca-109 cells (3 × 10^5^) treated with 1640 medium without FBS were plated to the upper chamber. 1640 medium with the supernatant of NIH3T3 cells as chemoattractants were plated in the lower chamber of the 24-well pates. After incubation for 24 h, noninvading cells were removed mechanically from the upper chamber using a cotton swab. Cells that invaded to the lower surface of the transwell membrane were fixed in methanol for 30 min at 37°C and stained with 0.05% crystal violet for 1 h. Cells were quantified by counting the number of stained cells in five individual fields by microscopy. The experiments were performed independently four times.

### Statistical analysis

SPSS17.0 was used for statistical analysis. One-way analysis of variance (ANOVA) and the χ2 test were used to analyze the significance between groups. Multiple comparisons between the parental and control vector groups were made using the Least Significant Difference test when the probability for ANOVA was statistically significant. All data represent mean ± SD. Statistical significance was set at *p* < 0.05.

## Results

### STAT5 siRNA inhibited significantly the mRNA and protein expression of STAT5、Bcl-2 and Cyclin D1

**Figure 1:**
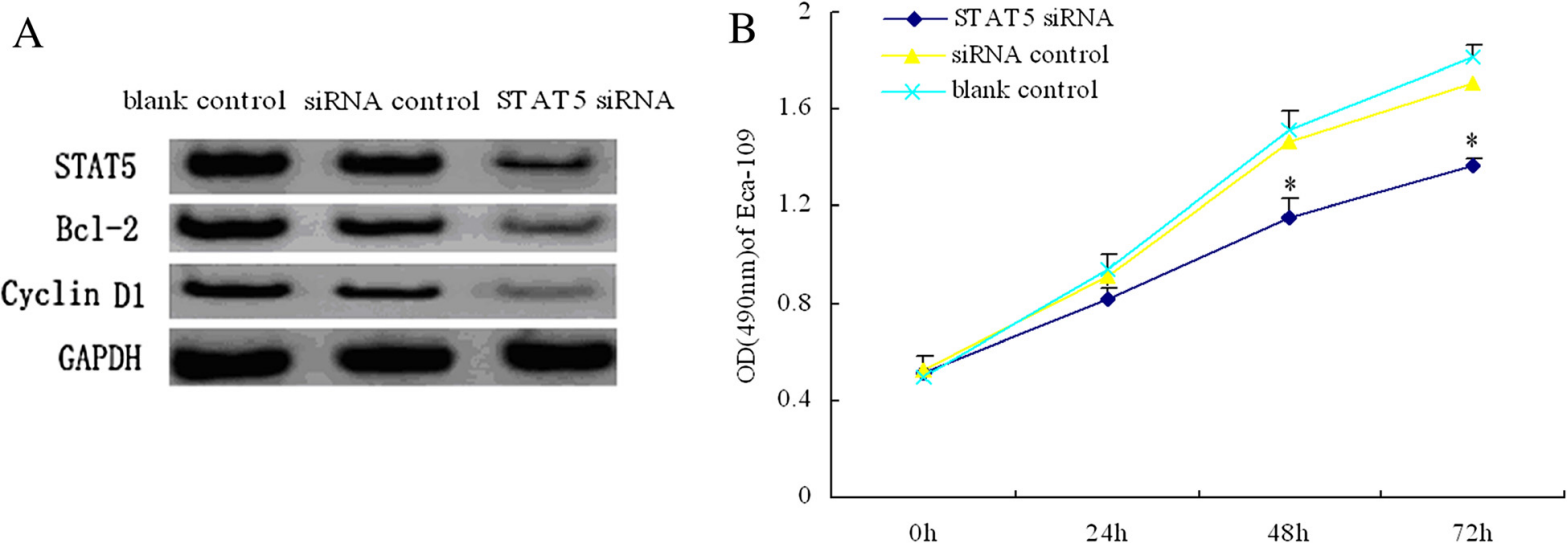
**The results of protein expression and cell proliferation. A** STAT5 siRNA inhibited significantly the protein expression of STAT5、Bcl-2 and Cyclin D1. Cells were incubated with different synthetic oligonucleotides as described in the materials and methods section, and CXCR4 protein was quantified by Western-Blot. The targeted CXCR4 siRNA inhibited significantly the protein expression of STAT5、Bcl-2 and Cyclin D1 gene. **B** cell proliferation was assessed using the MTT assay. Data are presented as the mean of four experiments. Silencing the STAT5 depresses the proliferation of esophageal carcinoma cell line Eca-109. Significant difference (*p* <0.05).

The result of RT-PCR was in the Table 1. The STAT5、Bcl-2 and Cyclin D1 mRNA expression in untransfected Eca-109 cells were coincident with that in the cells transfected with control vector( pRNAT-U6.1/Neo-siCon). The statistical data between them was not significance (p > 0.05). RT-PCR results showed that STAT5、Bcl-2 and Cyclin D1 mRNA expression in STAT5 siRNA group were significantly inhibited compared to siRNA control and blank control group as shown in Table 2. The targeted STAT5 siRNA inhibited significantly the mRNA expression of STAT5 gene. The results of Western-Blot showed the expression of STAT5 protein was significantly decreased in Eca-109 cells in STAT5 siRNA group (Figure 1A). The result showed that the Bcl-2 and Cyclin D1 expression in Eca-109 cells transfected with STAT5 siRNA vector were significantly decreased.

**Table 2:** STAT5 siRNA inhibited significantly the mRNA expression of STAT5、Bcl-2 and Cyclin D1

**Group**	**n**	**STAT5/GAPDH**	**Bcl-2/GAPDH**	**Cyclin D1/GAPDH**
STAT5 siRNA	4	0.301 ± 0.012*	0.266 ± 0.012*	0.048 ± 0.005*
siRNA control	4	0.836 ± 0.038	0.645 ± 0.023	0.261 ± 0.013
The blank control	4	0.857 ± 0.041	0.687 ± 0.027	0.273 ± 0.012

Cells were incubated with different synthetic oligonucleotides as described in the materials and methods section, and STAT5、Bcl-2 and Cyclin D1mRNA were quantified by real time PCR. The targeted STAT5 siRNA inhibited significantly the expression of STAT5、Bcl-2 and Cyclin D1 gene (*p <0.05).

### STAT5 siRNA inhibited esophageal carcinoma cells proliferation in vitro

Compared with untransfected cells and cells transfected with control vector, the Eca-109 cell transfected with vector (pRNAT-U6.1/Neo-siSTAT5) were significantly depressed respectively at 24 h、48 h and 72 h. The cells become round at 24h. With the longer time, the smaller shed cells become more and more. The result showed that silencing the STAT5 depresses the proliferation of esophageal carcinoma cell line Eca-109 (Figure 1B).

### STAT5 siRNA affect the cell cycle of Eca-109 cells

Compared with untransfected Eca-109 cells and cells transfected with control vector, The proportion of S and G2/M period frequency of Eca-109 cell transfected with vetor (pRNAT-U6.1/Neo-siSTAT5) was significantly decreased(*p* < 0.05). The proportion of G0/G1 period frequency was significantly increased (*p* < 0.05) as shown in Table 3.

**Table 3:** STAT5 siRNA affect the cell cycle of Eca-109 cells

**Group**	**G**_**0**_ **~ G**_**1**_**(%)**	**S(%)**	**G**_**2**_ **~ M(%)**
STAT5 siRNA	75.9 ± 2.3	20.85 ± 0.55*	3.25 ± 0.12*
siRNA control	64.23 ± 1.92	27.8 ± 0.61	7.96 ± 0.27
The blank control	64.69 ± 2.16	26.46 ± 0.59	8.85 ± 0.31

The cell cycle was detected by flow cytometry. The proportion of S and G2/M period frequency of Eca-109 cell transfected with vetor (pRNAT-U6.1/Neo-siSTAT5) was significantly decreased (*p < 0.05). The proportion of G0/G1 period frequency was significantly increased (*p < 0.05).

### STAT5 siRNA induced the apoptosis and suppressed invasion and metastasis of Eca-109

Compared with untransfected Eca-109 cells and cells transfected with conrtrol vector, The average apoptosis rate of Eca-109 cell transfected with vector (pRNAT-U6.1/Neo-siSTAT5) was significantly increased (*p* < 0.05),The average amount of cells penetrating matrigel was significantly decreased(*p* < 0.05). The result showed that silencing the STAT5 induced the apoptosis and suppressed invasion and metastasis of esophageal carcinoma cell line Eca-109 as shown in Table 4.

**Table 4:** STAT5 siRNA induced the apoptosis and suppressed invasion and metastasis of Eca-109

**Group**	**Transfected vector**	**The cell apoptosis rate (%)**	**The average amount of cells per field**
STAT5 siRNA	pRNAT-U6.1/Neo -siSTAT5	13.38 ± 1.87*	24.2 ± 5.1*
siRNA control	pRNAT-U6.1/Neo-siCon	4.76 ± 0.69	65.2 ± 10.4
The blank control	-	4.49 ± 0.73	71.9 ± 12.4

The average apoptosis rate of Eca-109 cell transfected with STAT5 siRNA was significantly increased (*p < 0.05), The average amount of cells penetrating Matrigel was significantly suppressed (*p < 0.05).

## Discussion

The activation of JAK phosphorylates STAT proteins, leading to their dimerization and translocation into the nucleus [15]. In the nucleus, STATs act as transcription factors with pleiotropic downstream effects. STATs are phosphorylated on tyrosine residues via JAK kinases and on serine residues by a variety of serine/threonine kinases [16]. STATs then dimerize, translocate to the nucleus and bind DNA, initiating the transcription of target genes. STAT proteins mediate cell growth, differentiation, apoptosis, transformation, and other functions. In cancer cells, STAT5 activation lead to the increased expression of downstream target genes (Bcl-xL, mcL-1, cyclinD1 /D2 and c-myc), which increased cell proliferation, cell survival, angiogenesis, and immune system evasion [17]. STAT5 is very mportant in STATs family, including STAT5a and STAT5b, which plays an important role in many cancers [18].

The study designed and constructed the siRNA vector (pRNAT-U6.1/Neo -siSTAT5) aiming to STAT5. Then it was transfected into Eca-109 cells. The result of RT-PCR and Western-blot demonstrated STAT5 was significantly suppressed, not only the mRNA expression, but also the protein expression, The Bcl-2 and Cyclin D1 expression in Eca-109 cells transfected with STAT5 siRNA vector were significantly decreased. The result of MTT demonstrated the cell growth was significantly suppressed. The result of flow cytometry demonstrated the proportion of S and G2/M period frequency was significantly decreased (*p* < 0.05). The proportion of G0/G1 period frequency was significantly increased (*p* < 0.05). Zhao Zhengjun e.tal approved silencing the STAT5 of liver carcinoma cell SMMC27721 induced the cell apoptosis,using siRNA [19]. Duan zhao e.tal approved silencing the STAT5 depressed the proliferation of cervical carcinoma cell HeLa and induced the cell apoptosis [20]. The proportion of S and G2/M period frequency was significantly decreased. The proportion of G0/G1 period frequency was significantly increased. All are associated with our study. There are other risk factors associated with esophageal carcinomas. Fascin induces membrane protrusions and cell motility [21]. Fascin overexpression plays a role in tumor growth and progression in ESCC and that cell death caused by its downregulation might be induced by cell adhesion loss [22]. This indicates that targeting fascin pathway could be a novel therapeutic strategy for the human ESCC. ATP-binding cassette sub-family G member 2 (ABCG2) is a protein that in humans is encoded by the ABCG2 gene. ABCG2 participates in efflux of many chemotherapeutic agents [23]. ABCG2 is often expressed in hematopoietic progenitor or stem cells. Vacuolar-H + −ATPase (V-ATPase) plays a key role in adjusting and maintaining intracellular pH and in regulating the drug tolerance of cells [24]. Both ABCG2 and V-ATPase were over-expressed in esophageal squamous cancer cells. Their expression was associated with pathological grade, TNM stage and tumor metastasis in esophageal squamous cancer cells [25]. ABCG2 and V-ATPase expression may be strongly associated with drug resistance and tumor metastasis. All the risk factors were very important in the occurrence and development of esophageal carcinomas.

The study first approved silencing the STAT5 of esophageal carcinoma cell line Eca-109 induced the apoptosis and supressed the proliferation,invasion and metastasis, which indicated STAT5 might be a novel therapeutic strategy for the human ESCC.

## Conclusions

STAT5 silenced by siRNA could induce the apoptosis, suppress the proliferation、invasion and metastasis of esophageal carcinoma cell line Eca-109, which indicated STAT5 might be a novel therapeutic strategy for the human ESCC.

## Competing interests

The authors declare that they have no competing interests.

## Authors’ contribution

QY, ML and HX: conceived of the study, and participated in its design and coordination and helped to draft the manuscript. WQZ and GQZ: carried out part of experiments and wrote the manuscript. LPP and TW performed the statistical analysis. All authors read and approved the final manuscript.
